# Dr. Peter Failing

**Published:** 1902-03

**Authors:** 


Dr. Peter Failing, of Gasport, N.Y., died suddenly in his office November 23, 1901, aged 69 years.

